# Impact of Pre-Existing Urinary Antimicrobial Agents on Culture Yield, Diagnostic Accuracy, and the Detection of Significant Bacteriuria in Community-Acquired Urinary Tract Infections

**DOI:** 10.7759/cureus.84038

**Published:** 2025-05-13

**Authors:** Vivekanand B Jadhav, Sanjo Gupta, Arundhuti Paul, Rahul Bhalsinge, Ritu Bhatnagar, Savita V Jadhav

**Affiliations:** 1 Microbiology, Pacific Medical College and Hospital (Pacific Medical University), Udaipur, IND; 2 Microbiology, Institute of Liver and Biliary Sciences, New Delhi, IND; 3 Pharmacology, LN Medical College and JK Hospital, Bhopal, IND; 4 Medical Microbiology and Diagnostic Microbiology, Pacific Medical College and Hospital (Pacific Medical University), Udaipur, IND

**Keywords:** antimicrobial agents, antimicrobial sensitivity tests, diagnosis drug resistance, significant bacteriuria, urinary tract infections

## Abstract

Introduction: Pre-existing antibacterial substances (ABS) in urine can significantly impact the diagnostic and therapeutic management of urinary tract infections (UTIs) by altering microbial culture outcomes and influencing the detection and resistance profiles of uropathogens. The presence of pre-existing ABS may lead to false-negative or insignificant culture results, affecting clinical decision-making. This study aims to assess the impact of pre-existing ABS on culture positivity, microbial distribution, and antibiotic susceptibility patterns in outpatients, particularly those with prior antimicrobial exposure.

Methods: A prospective observational study was conducted at a tertiary care hospital on 621 outpatients with clinical symptoms suggestive of UTI. Urine samples were analyzed for pre-existing ABS and classified into pre-existing ABS-positive (n = 158) and pre-existing ABS-negative (n = 463) groups. Data on demographics, clinical characteristics, comorbidities, and microbiological profiles were collected. Associations between pre-existing ABS presence and variables such as prior antibiotic exposure, requisition form completeness, and diabetes mellitus were evaluated using chi-square tests. A p-value of <0.05 was considered statistically significant.

Results: The study population comprised 356 females (57.32%), with a mean age of 30.68 years. The most common presenting symptom was an increased urinary frequency (76.16%), followed by abdominal pain (62.47%). Pre-existing ABS were detected in 158 (25.44%) cases. Significant associations were observed between pre-existing ABS presence and prior antibiotic use (χ² = 31.61 *p < 1.88 × 10⁻⁸,),* adequate requisition form documentation (*χ² (df) = 40.58, p < 1.88 × 10⁻¹⁰*), and diabetes mellitus (*χ² (df) = 9.02, p = 0.0027).* Culture positivity was significantly reduced in pre-existing ABS-positive samples, with lower isolation rates of *Escherichia coli* (*27.21% vs. 39.52%, p = 0.007*) and an increased prevalence of *Proteus mirabilis* (*10.75% vs. 4.96%, p = 0.018*). Pre-existing ABS-positive samples also showed significantly higher rates of no growth (*17.49% vs. 9.49%, p = 0.023*) and insignificant growth (*19.62% vs. 12.00%, p = 0.026*). Antibiotic susceptibility analysis revealed that pre-existing ABS-positive isolates of *E. coli* and *Klebsiella pneumoniae* exhibited reduced susceptibility to amoxicillin-clavulanic acid, cefazolin, and imipenem compared to pre-existing ABS-negative isolates, suggesting a potential impact of prior antimicrobial exposure on resistance patterns.

Conclusion: The presence of pre-existing ABS in urine significantly alters microbial culture outcomes, leading to reduced detection of common uropathogens and an increased likelihood of false-negative or insignificant culture results. Additionally, pre-existing ABS-positive isolates demonstrate higher resistance to key antibiotics, highlighting the need for careful interpretation of urine culture results in patients with prior antimicrobial exposure. Comprehensive documentation of antibiotic history and requisition form completeness is essential to improve diagnostic accuracy. Further research is warranted to explore the role of pre-existing ABS in promoting antimicrobial resistance in community-acquired UTIs and to refine diagnostic criteria for significant bacteriuria in the presence of pre-existing ABS.

## Introduction

Urinary tract infections (UTIs) are among the most prevalent bacterial infections worldwide, accounting for approximately 150 million cases annually, and are a leading cause of outpatient antimicrobial prescriptions [1,2]. The burden of UTIs is particularly pronounced in low- and middle-income countries (LMICs), where diagnostic limitations and unrestricted access to antibiotics contribute to suboptimal management and increased antimicrobial resistance (AMR) [3]. *Escherichia coli* (*E. coli*) remains the predominant uropathogen, followed by *Klebsiella pneumoniae* (*K. pneumoniae*), *Proteus mirabilis* (*P. mirabilis*), *Enterococcus faecalis* (*E. faecalis*), and *Staphylococcus saprophyticus* (*S. saprophyticus*), with many strains now exhibiting multidrug resistance (MDR) phenotypes [4-6].

AMR in UTIs is driven by factors such as empirical antimicrobial use without laboratory confirmation, over-the-counter antibiotic access, and the misuse of broad-spectrum agents, often in the absence of microbiological guidance [7]. A crucial, yet often underrecognized factor complicating UTI diagnosis is the presence of pre-existing antimicrobial substances (ABS) in urine, defined as residual antibiotics or other antibacterial agents in a patient’s urine sample that are present due to prior antibiotic consumption, self-medication, or inadvertent exposure [8-10]. These substances can suppress or inhibit bacterial growth during in vitro culture, leading to false-negative results despite underlying infection [11]. Such diagnostic interference compromises the detection of significant bacteriuria, typically defined as ≥10⁵ colony-forming units (CFU)/mL in a properly collected midstream urine sample and undermines the diagnostic sensitivity of urine culture, which remains the gold standard [12].

Recent studies have reported ABS detection rates in urine ranging from 10% to 30% in outpatient and hospital settings, with notable implications for microbiological diagnostics and antimicrobial stewardship [13-15]. In particular, culture-negative, symptomatic UTI cases are increasingly attributed to pre-existing ABS, resulting in delayed or inappropriate treatment, overestimation of non-bacterial causes, and missed opportunities for AMR surveillance [16,17]. In LMICs, where access to antibiotics without prescription is common and diagnostic infrastructure may be limited, this issue is exacerbated and remains underreported in national and regional AMR databases [1,3-6].

Given these challenges, integrating ABS detection into routine diagnostic workflows is essential for accurate interpretation of urine culture results and rational antimicrobial use. Approaches such as the Urine Antibacterial Substance Assay (UABA) typically involve the use of reference indicator strains (*Escherichia coli* ATCC 25922) and have shown promise in detecting inhibitory substances in urine samples. These assays, when interpreted alongside detailed medication histories, can help identify cases where ABS may suppress bacterial growth in culture, thereby avoiding false-negative results and improving clinical decision-making [10-13].

This study aims to evaluate the prevalence of pre-existing antimicrobial substances in outpatient urine samples submitted for UTI diagnosis and assess their impact on significant bacteriuria and culture outcomes. The findings will inform the development of evidence-based diagnostic and stewardship strategies to enhance the detection and management of community-acquired UTIs, particularly in AMR-endemic regions.

## Materials and methods

Study design and location

This prospective observational study was conducted in the Department of Microbiology at a teaching tertiary care hospital for over one year. The study adhered to ethical guidelines, with informed consent obtained from all participants.

Sample size

A total of 621 patients presenting to the outpatient department (OPD) with symptoms suggestive of UTI were included.

Sample size calculation

The sample size was determined based on data from a pilot study of 621 participants, which provided estimates of prevalence and variability in culture yield and diagnostic accuracy. Using a global prevalence of 25% for significant bacteriuria in catheter-associated urinary tract infections (CA-UTIs) and aiming for a 95% confidence level (α = 0.05) and 80% power, the required sample size per group was calculated to be 289 participants. This was rounded up to 310 participants per group to account for potential data loss, resulting in a total sample size of 621 participants. This ensures adequate statistical power to detect significant differences between groups with pre-existing antimicrobial treatment and those without.

Inclusion criteria

The study included patients presenting with clinical symptoms indicative of UTI, specifically dysuria, increased urinary frequency, urgency, and lower abdominal pain. Eligible patients were referred for microbiological investigation, including urine culture and sensitivity testing, to confirm the diagnosis of UTI.

Exclusion criteria

Patients who developed hospital-acquired infections (HAIs) after 48 hours of hospital admission were excluded from the study. This criterion was established to focus on community-acquired infections, thereby minimizing confounding variables associated with nosocomial pathogens and hospital environments, which often exhibit different resistance patterns.

Sample collection and processing

Upon recruitment, patients received comprehensive instructions for collecting midstream urine specimens, emphasizing sterile techniques to reduce contamination risk. Urine samples were collected in sterile containers and transported promptly to the microbiology laboratory for analysis, adhering to established guidelines [1,13-15]

Gross examination

Urine samples underwent visual inspection for parameters such as color, turbidity, and visible particles or debris, which can indicate infection or sample contamination.

Microscopy

Wet mount preparations of urine samples were analyzed under light microscopy for the presence of pus cells (WBCs), red blood cells (RBCs), epithelial cells, and bacteria. The presence of five or more pus cells in the wet mount was taken as an indicator of significant bacteriuria [15]. Additionally, the examination included the identification of specific structures such as casts, crystals, yeast cells, and ova of Schistosoma haematobium. Bacterial motility was also assessed to aid in the identification of potential pathogens. Microscopic findings, alongside gross examination, contribute significantly to the preliminary diagnostic process, guiding further culture-based investigations [15-17].

Urine antibacterial substance assay 

In the Urine Antibacterial Substance Assay (UABA), two Mueller-Hinton agar plates were employed for testing each bacterial isolate. Lawn cultures of *Escherichia coli* ATCC® 25922™ and *Staphylococcus aureus* ATCC® 25923™ were inoculated onto separate plates. A sterile cotton swab dipped into 0.5 McFarland standard bacterial suspensions of the isolated strains was rotated three times at 60° intervals on each plate to achieve uniform inoculation. 6 mm sterile filter paper discs (Whatman grade 1) were prepared and sterilized by autoclaving. These discs were placed onto the inoculated agar surface with a 10-12 mm distance between each disc. A loopful (0.01 ml/1 μl) of the urine sample was then applied to the center of the corresponding discs. The plates were incubated overnight at 35-37°C. After incubation, the presence of a clear zone of inhibition around the disc was considered indicative of antimicrobial activity (UABA-positive), while the pre-existing ABS of such a zone was interpreted as UABA-negative, signifying no detectable antibacterial activity in the urine sample [17-19].

Urine culture, identification of uropathogens, and antibiotic susceptibility testing

A urine culture was performed using standard microbiological techniques to identify potential uropathogens. Freshly collected midstream urine samples were inoculated onto MacConkey agar and blood agar plates using a calibrated loop and incubated at 35-37°C for 24 to 48 hours. After incubation, colony growth was assessed, with a threshold of ≥10⁵ colony-forming units (CFU)/ml considered significant for bacteriuria, indicating a probable urinary tract infection (UTI). This threshold is widely accepted in clinical practice, balancing sensitivity and specificity for UTI diagnosis. The identification of uropathogens was conducted using conventional biochemical methods, and further confirmation was made using the VITEK 2C automated system, where necessary, to ensure precise organism identification [1,3,13,15-17].

Antibiotic susceptibility testing

Antibiotic susceptibility testing (AST) was performed using the Kirby-Bauer disk diffusion method by Clinical and Laboratory Standards Institute (CLSI) guidelines (CLSI, 2022). Additionally, minimum inhibitory concentrations (MICs) were determined using the VITEK 2C system to enhance accuracy in resistance profiling. A comprehensive antibiotic panel, including beta-lactams, fluoroquinolones, aminoglycosides, and other relevant antimicrobial classes, was tested to assess resistance patterns. These susceptibility profiles are essential for guiding empirical therapy and addressing the growing concern of antimicrobial resistance (AMR) in uropathogens [15-17].

Ethics approval

The study was conducted using anonymized clinical urine samples collected as part of routine diagnostic procedures, without any direct patient contact or use of personally identifiable information. Ethical approval was obtained from the Institutional Ethics Committee of LNCT Medical College, Indore (Approval No.: VU/LNCTMC/IEC/2024/14). All procedures were performed in accordance with the ethical standards of the institutional committee and relevant national guidelines and regulations.

Statistical analysis

Statistical analysis was performed using IBM Corp. Released 2012. IBM SPSS Statistics for Windows, Version 21.0. Armonk, NY: IBM Corp. The McNemar test was used to assess paired categorical data. A p-value of <0.001 was obtained for the urine antibacterial substance assay (UABA), indicating a statistically significant association.

## Results

This study analyzed the demographic profile, clinical presentation, pre-existing antimicrobial substance (ABS) status, and co-morbid conditions of 621 patients presenting with suspected urinary tract infections (UTIs). The findings provide insights into age and gender distribution, symptomatology, and underlying health conditions that may influence cultural positivity and resistance patterns.

Age distribution

The study population comprised 621 individuals, stratified into seven age groups. The highest proportion of patients, 194 (31.23%), belonged to the 21-30 years age group, followed by 127 (20.45%) in the ≤10 years group. Patients aged 31-40 years accounted for 102 (16.42%), while 73 (11.75%) were in the 41-50 years category. The elderly population, those aged >60 years, comprised 28 (4.5%), and the 51-60 years group represented 45 (7.24%). A smaller (six) subset of patients, 52 (8.37%), fell within the 11-20 years age group. The mean age of the study population was 30.68 years, indicating a relatively young cohort (Table 1).

**Table 1:** Comprehensive demographic, clinical profile, pre-existing ABS status, and co-morbidities among study cases (n = 621) ABS: Antibacterial substances

Variables	Categories	Number of Cases (N = 621) (%)
Age (years)	≤ 10	127(20.45)
	11 – 20	52(8.37)
	21 – 30	194(31.23)
	31 – 40	102(16.42)
	41 – 50	73(11.75)
	51 – 60	45(7.24)
	> 60	28(4.5)
	Mean Age	30.68
Gender	Male	265(42.67)
	Female	356(57.32)
Signs and symptoms	Increased Urinary Frequency	473(76.16)
	Abdominal Pain	388(62.47)
	Fever with Chills	183(29.46)
	Dysuria	92(14.81)
	Haematuria	70(11.27)
Pre-existing ABS status	PRE-EXISTING ABS	158(25.44)
	Non-PRE-EXISTING ABS	463(74.55)
Co-morbidities	Obstructive Pathology	203(32.68)
	Diabetes Mellitus	64(10.30)
	Pregnancy	14(2.25)

Table 1 summarizes the demographic characteristics, clinical presentation, pre-existing ABS status, and co-morbidities in 621 study cases.

Age and gender distribution

The mean age of the study population was 30.68 years, with the highest proportion of cases observed in the 21-30-year age group, accounting for 194 cases (31.23%). Females constituted a greater proportion, with 356 cases (57.32%), consistent with the well-documented higher prevalence of UTIs in women. This disparity is attributed to anatomical and physiological factors, including a shorter urethra and hormonal influences, which increase susceptibility to ascending infections.

Clinical presentation

Increased urinary frequency was the most commonly reported symptom, present in 473 cases (76.16%), followed by abdominal pain in 388 cases (62.47%) and fever with chills in 183 cases (29.46%). Dysuria and hematuria were observed in 92 cases (14.81%) and 70 cases (11.27%), respectively, and though less frequent, they indicate significant urinary tract pathology. The predominance of these symptoms highlights the varied clinical spectrum of UTIs and the necessity for targeted diagnostic and therapeutic approaches.

Pre-existing ABS status

Among the 621 cases, pre-existing ABS was identified in 158 cases (25.44%), suggesting prior antimicrobial exposure or persistent colonization, whereas 463 cases (74.55%) were negative for pre-existing ABS. This finding reinforces the importance of assessing antimicrobial history in culture-based diagnosis to distinguish true infections from colonization, thereby preventing unnecessary antimicrobial use.

Co-morbidities in pre-existing ABS cases

Obstructive pathology was the most frequently observed co-morbidity among pre-existing ABS cases, reported in 203 cases (32.68%), indicating its role in recurrent or persistent infections due to impaired urinary flow. Diabetes mellitus, a well-established risk factor for UTIs due to immune dysfunction and glycosuria, was present in 64 cases (10.30%). Pregnancy, a recognized predisposing factor for UTIs due to hormonal changes and ureteral dilatation, was observed in 14 cases (2.25%).

Clinical implications

These findings underscore the significant influence of patient demographics, clinical history, and co-morbidity on UTI management. The high prevalence of pre-existing ABS highlights the necessity for prudent antimicrobial stewardship to enhance diagnostic accuracy, prevent overtreatment, and improve clinical outcomes. A comprehensive evaluation of risk factors, coupled with microbiological confirmation, is essential for optimizing UTI management strategies.

Table 2 presents the association between pre-existing ABS and key clinical variables, including prior antibiotic exposure, the adequacy of requisition form information, and the presence of diabetes mellitus. Among the 621 cases analyzed, 158 (25.44%) exhibited pre-existing ABS. A significantly higher proportion of pre-existing ABS cases had a history of prior antibiotic use (41.44%, χ² = 31.61, p < 0.0001) compared to those without pre-existing ABS. Similarly, adequate requisition form information was more frequently observed among pre-existing ABS cases (58.78%, χ² = 40.58, p < 0.0001), suggesting that well-documented clinical details may facilitate the identification of persistent bacteriuria. The presence of diabetes mellitus was also significantly associated with pre-existing ABS, with 64.06% of diabetic patients showing pre-existing ABS positivity (χ² = 9.02, p = 0.0027). These findings highlight the critical role of prior antimicrobial exposure, clinical documentation, and underlying comorbidities in the persistence of bacteriuria, underscoring the importance of antimicrobial stewardship and comprehensive patient evaluation in UTI management.

**Table 2:** Association of antibiotic history, requisition form information, and diabetes mellitus with the presence of pre-existing ABS: Chi-square analysis

Variable	Category	No. of Cases (N = 621)	Pre-existing ABS (n = 158) [n, %]	Non-pre-existing ABS (n = 463) [n, %]	Chi-square (χ²) Value	p-value
Antibiotic consumption history	Yes	152	63 (41.44%)	89 (58.55%)	31.61	<0.0001
	No	469	87(18.55%)	372 (79.74%)		
Adequate Information on Requisition forms	Yes	148	87 (58.78%)	61 (41.21%)	40.58	<0.0001
	No	473	71 (15.01%)	402 (84.98%)		
Diabetes mellitus	Yes	64	41 (64.06%)	23 (35.93%)	9.02	0.0027

Table 3 presents the distribution of bacterial and fungal isolates among cases of pre-existing ABS (N = 158) and non-pre-existing ABS (N = 463). Among pre-existing ABS cases, the most frequently isolated pathogen was *E. coli*, with 43 cases (27.21%), followed by *K. pneumoniae*, with 23 cases (14.55%), and *P. mirabilis*, with 17 cases (10.75%). The prevalence of *E. coli* was significantly higher in non-pre-existing ABS cases, with 183 cases (39.52%, p = 0.0073), suggesting a stronger association with community-acquired infections.

**Table 3:** Distribution of organisms in pre-existing ABS and non-pre-existing ABS cases

Organism (n)	Pre-existing ABS Cases (N = 158) [n, %]	Non-pre-existing ABS Cases (N = 463) [n, %]	p-value	Statistical Significance (p < 0.05)
No Growth (96)	15 (9.49%)	81 (17.49%)	0.0229	Yes
Insignificant Growth (87)	31 (19.62%)	56 (12.09%)	0.0264	Yes
Polymicrobial Flora (27)	10 (6.32%)	17 (3.67%)	0.2347	No
*E. coli *(213)	43 (27.21%)	183 (39.52%)	0.0073	Yes
*K. pneumoniae *(78)	23 (14.55%)	55 (11.87%)	0.4605	No
*P. mirabilis *(40)	17 (10.75%)	23 (4.96%)	0.0176	Yes
*Enterococcus faecalis *(31)	13 (8.22%)	18 (3.88%)	0.0510	No
*P. aeruginosa *(21)	8 (5.00%)	13 (2.80%)	0.2716	No
*Coagulase-negative Staphylococcus (CoNS) *(16)	7 (4.43%)	9 (1.94%)	0.1578	No
*Yeast *(12)	4 (2.53%)	8 (1.72%)	0.7649	No

A significantly higher proportion of culture-negative results was observed in non-pre-existing ABS cases, with 81 cases (17.49%) compared to 15 cases (9.49%) in pre-existing ABS cases (p = 0.0229), indicating the potential impact of prior antimicrobial exposure on bacterial recovery. Conversely, insignificant growth was more frequent in pre-existing ABS cases, with 31 cases (19.62%) compared to 56 cases (12.09%) in non-pre-existing ABS cases (p = 0.0264), reinforcing the role of antimicrobial history in influencing microbiological outcomes.

The presence of *P. mirabilis* was significantly associated with pre-existing ABS, with 17 cases (10.75%) compared to 23 cases (4.96%) in non-pre-existing ABS cases (p = 0.0176), underscoring its role in recurrent or persistent infections, potentially linked to urinary stone formation and biofilm production. Other organisms, including *E. faecalis* with 13 cases (8.22%) vs. 18 cases (3.88%, p = 0.0510), *P. aeruginosa* with 8 cases (5.00%) vs. 13 cases (2.80%, p = 0.2716), coagulase-negative *staphylococci* (CoNS) with 7 cases (4.43%) vs. 9 cases (1.94%, p = 0.1578), and *Candida spp.* with 4 cases (2.53%) vs. 8 cases (1.72%, p = 0.7649), did not exhibit statistically significant differences between groups.

These findings highlight the importance of incorporating prior antimicrobial exposure into clinical decision-making for bacteriuria management. The significant differences in pathogen distribution between pre-existing and non-pre-existing ABS cases underscore the need for prudent antimicrobial stewardship to improve diagnostic accuracy and therapeutic interventions.

Table 4 compares the antimicrobial susceptibility profiles of *E. coli* and *K. pneumoniae* isolated from urine specimens stratified by the presence or absence of pre-existing ABS. The analysis includes 43 *E. coli* and 23 *K. pneumoniae* isolates from the pre-existing ABS-positive group and 170 E. coli and 55 *K. pneumoniae* isolates from the pre-existing ABS-negative group.

**Table 4:** Antimicrobial susceptibility patterns of E. coli and K. pneumoniae in pre-existing and non-pre-existing AB cases Ceftazidime-clavulanic acid was tested specifically for strains resistant to ceftazidime (ESBL producers’ phenotypic detection), while imipenem combined with EDTA was tested exclusively for strains resistant to imipenem (metallo-beta-lactamase (MBL) producers’ phenotypic detection).

Antimicrobial	Susceptibility In Pre-Existing ABS Patients [n=158]		Susceptibility In Non-Pre- Existing ABS Patients [n=463]	
	*E. coli *(n = 43)%	*K. pneumoniae* (n = 23)	*E. coli *(n = 170)	*K. pneumoniae *(n = 55)
Amoxicillin-Clavulanic Acid	16 (37.20)	11(47.83)	91(53.53)	32 (58.18)
Cotrimoxazole	24 (55.81)	13(56.52)	102 (60)	36 (65.45)
Nitrofurantoin	30 (69.76)	16(69.57)	143(84.12)	42 76.36)
Cefazolin	15 (34.88)	9 (39.13)	96 (56.47)	30(54.54)
Amikacin	26 (60.46)	14 (60.87)	138 (81.18)	38 (69.09)
Gentamicin	19 (44.18)	8 (34.78)	103 (60.59)	36 (65.45)
Ceftriaxone	18 (41.86)	11 (47.83)	119 (70)	32 (58.18)
Ceftazidime	15 (34.88)	13 (56.52)	121(71.81)	46 (83.63)
Ceftazidime-Clavulanic Acid	28 (65.11)	10 (43.47)	62 (36.47)	9 (16.36)
Ampicillin-Sulbactam	16 (37.20)	8 (34.78)	91(53.53)	40 (72.72)
Imipenem	32(74.41)	16(69.57)	168 (98.82)	49(89.09)
Imipenem +EDTA	11 (25.58)	7 (30.43)	15 (8.82)	6 (10.90)
Piperacillin-Tazobactam	15 (34.88)	10 (43.48)	115 (67.65)	34 (61.81)
Meropenem	28 (65.11)	14 (60.87)	159 (93.53)	38 (69.09)
Colistin	43 (100)	23 (100)	170 (100)	55 (100)
Tetracycline	21 (48.83)	12 (52.17)	121(71.28)	35 (63.63)
Tigecycline	43 (100)	23 (100)	170 (100)	55 (100)
Ciprofloxacin	26 (60.46)	13 (56.52)	142 (83.53)	37 (67.27)
Norfloxacin	29 (67.44)	14 (60.87)	137 (80.59)	40 (72.72)
Nalidixic Acid	20 (46.51)	11 (47.83)	125 (73.53)	42 (76.36)

Overall, isolates recovered from urine specimens with pre-existing ABS demonstrated consistently lower susceptibility to a broad spectrum of antimicrobial agents, including β-lactams, aminoglycosides, and fluoroquinolones. This trend is particularly pronounced in key therapeutic agents such as imipenem, meropenem, amikacin, and ceftriaxone. For example, imipenem susceptibility in *E. coli* was 32 (74.41%) in the pre-existing ABS group versus 168 (98.82%) in the non-ABS group, and 16 (69.57%) versus 49 (89.09%) in *K. pneumoniae*, respectively.

Ceftazidime-clavulanic acid and imipenem-EDTA combination testing, employed for phenotypic detection of extended-spectrum β-lactamase (ESBL) and metallo-β-lactamase (MBL) production, respectively, revealed significantly higher resistance among pre-existing ABS-positive isolates. Specifically, the proportion of isolates requiring ESBL confirmatory testing was markedly elevated in the pre-existing ABS group, with ceftazidime-clavulanic acid susceptibility observed in 28 (65.11%) of *E. coli* and 10 (43.47%) of *K. pneumoniae*, compared to only 62 (36.47%) and 9 (16.36%), respectively, in the non-ABS group. Similarly, resistance to imipenem and its reversal by EDTA was substantially more frequent in the pre-existing ABS group, 11 (25.58%) in *E. coli* and 7 (30.43%) in *K. pneumoniae* relative to the non-ABS group (15 (8.82%) and 6 (10.90%), respectively), indicating a higher prevalence of MBL-producing strains.

These findings are consistent with global literature, which reports that prior antimicrobial exposure exerts selective pressure that enriches for multidrug-resistant phenotypes, particularly ESBL and carbapenemase producers. The data underscore the clinical importance of detecting residual antimicrobials in diagnostic urine specimens, as their presence is strongly associated with elevated resistance rates and may compromise culture-based detection of significant bacteriuria. Integrating pre-existing ABS screening into routine diagnostics may thus enhance the accuracy of susceptibility profiling and guide more effective antimicrobial stewardship interventions.

**Figure 1:**
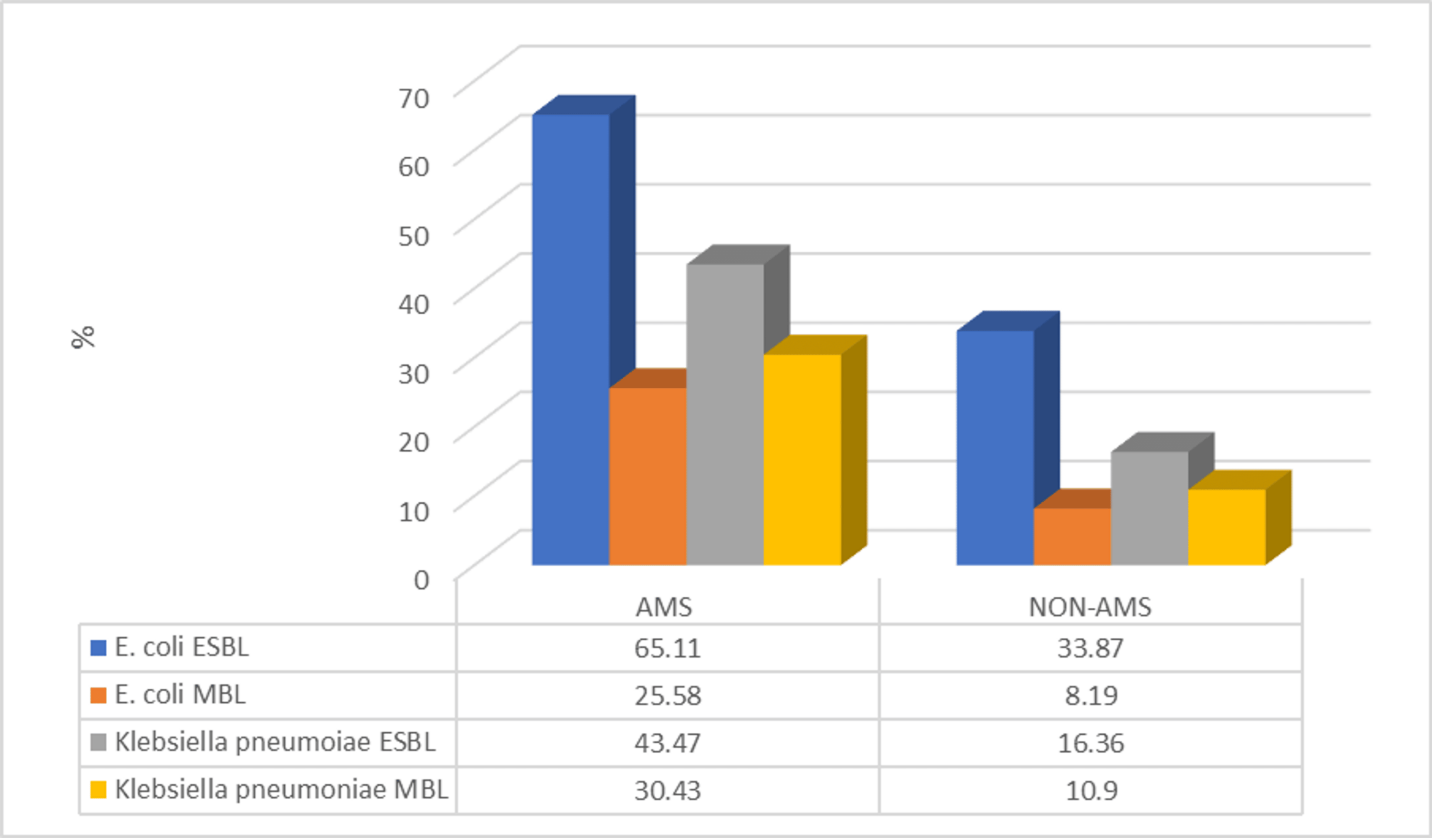
Prevalence of ESBL and MBL-producing E. coli and K. pneumoniae in patients with and without pre-existing ABS in urine

Figure 1 illustrates the prevalence of ESBL and MBL-producing *E. coli* and *K. pneumoniae* isolates among patients with detectable pre-existing antimicrobial substances (ABS) in urine and those without (non-ABS).

Among ESBL-producing *E. coli*, 28 out of 43 (65.11%) isolates were detected in AMS-positive patients, whereas 62 out of 183 (33.87%) isolates were found in non-AMS patients. Similarly, ESBL-producing *K. pneumoniae* was more frequent in AMS-positive cases, with 10 out of 23 (43.47%) isolates, compared to 9 out of 55 (16.36%) in non-AMS cases. These findings indicate a higher prevalence of ESBL producers among patients with pre-existing antimicrobial substances in urine.

For MBL-producing strains, imipenem-resistant *E. coli* tested with EDTA identified 11 out of 43 (25.58%) isolates in AMS-positive patients, whereas only 15 out of 183 (8.19%) were detected in non-AMS cases. Similarly, MBL-producing *K. pneumoniae* was found in seven out of 23 (30.43%) isolates in AMS-positive patients compared to six out of 55 (10.90%) in non-AMS cases. These results suggest that pre-existing antimicrobial substances in urine may contribute to a selective pressure, leading to an increased proportion of carbapenemase-producing organisms.

The increased prevalence of ESBL- and MBL-producing strains in AMS-positive cases underscores the need for targeted antimicrobial stewardship interventions to mitigate resistance development and optimize treatment strategies (Figure 1).

## Discussion

Impact of pre-existing antibacterial substances on diagnostic accuracy

This study highlights the critical influence of pre-existing ABS on the diagnostic accuracy of community-acquired urinary tract infections (CA-UTIs) and the associated AMR landscape. Sub-therapeutic concentrations of antibiotics, frequently resulting from undocumented or inappropriate prior use, may suppress microbial growth sufficiently to yield false-negative cultures or insignificant growth, thereby compromising clinical diagnosis [10-12]. Given the easy availability of over-the-counter antibiotics, especially in low- and middle-income countries, diagnostic workflows must be adapted to account for confounding factors [13].

Clinical and demographic characteristics

The patient cohort showed a female predominance (57.32%), consistent with established UTI epidemiology, attributing higher risk in women to anatomical and physiological factors [14]. The most frequent presenting symptoms, urinary frequency (76.16%), abdominal pain (62.47%), and febrile illness with chills (29.46%), are indicative of both lower and upper urinary tract involvement, whereas hematuria (11.27%) was relatively uncommon, suggesting limited uroepithelial damage [15,16].

Association of pre-existing ABS with antimicrobial resistance

Pre-existing ABS were detected in 25.44% of samples, with a significant association between prior antibiotic use and pre-existing ABS positivity (41.44% vs. 20.26%; χ² = 31.61, P = 1.88 × 10⁻⁸), reinforcing the role of pre-existing antimicrobial exposure in shaping resistance patterns [17]. Multidrug-resistant organisms (MDROs), including extended-spectrum β-lactamase (ESBL) and metallo-β-lactamase (MBL) producers, were significantly more prevalent in pre-existing ABS-positive samples, underscoring the selective pressure exerted by residual antibiotics in biological specimens [18].

Among *E. coli* isolates, 65.11% of ESBL-producing strains were from pre-existing ABS-positive patients, compared to 33.87% of ABS-negative individuals. Similarly, MBL-producing *E. coli* and *K. pneumoniae* strains were substantially more common in pre-existing ABS-positive cases (25.58% and 30.43%, respectively), reflecting the contribution of pre-existing antibiotics to resistance emergence [19].

Effect of comorbidities on resistance and ABS positivity

Patients with obstructive uropathy (32.68%) and diabetes mellitus (10.30%) showed higher rates of recurrent infections and AMR. In diabetics, ABS positivity was notably elevated (64.1% vs. 35.9%), suggesting a synergistic effect of metabolic dysfunction and antibiotic exposure on resistance selection [20,21]. Pregnancy, though a minor proportion (2.25%), was also associated with increased risk, likely due to physiological urinary stasis and immunomodulation [22].

Pathogen distribution and culture yield

*E. coli* remained the predominant pathogen across both pre-existing ABS groups, though its prevalence was significantly higher in pre-existing ABS-negative patients (39.5% vs. 27.2%, p = 0.0073), likely reflecting typical CA-UTI etiology in unexposed individuals. In contrast, *P. mirabilis* was significantly more frequent in pre-existing ABS-positive cases (10.8% vs. 5.0%, p = 0.0176), potentially indicating selection pressure from recurrent or inappropriate antibiotic use [23].

Other pathogens, such as *E. faecalis*, *P. aeruginosa*, coagulase-negative *staphylococci* (CoNS), and *Candida spp.*, were not significantly different between groups but may still reflect altered microbiota following antimicrobial exposure. Culture-negative results were significantly more common in ABS-negative patients (17.5% vs. 9.5%, p = 0.0229), while pre-existing ABS-positive samples yielded more insignificant growth (19.6% vs. 12.1%, p = 0.0264), indicative of partial microbial suppression rather than eradication [24-27].

Clinical documentation and stewardship implications

Completeness of clinical documentation was significantly associated with ABS detection. In 58.78% of pre-existing ABS cases, forms were adequately completed compared to only 15.01% in cases with poor documentation. Accurate recording of recent antibiotic use enhances laboratory interpretation and mitigates misdiagnosis or inappropriate antimicrobial selection. Integration of standardized requisition forms with fields for antibiotic history should be a key element of diagnostic stewardship.

Need for diagnostic advancements

The paradoxical reduction in “No Growth” results in pre-existing ABS-positive samples, despite compromised diagnostic sensitivity, highlighting the limitations of conventional culture. Residual antibiotic activity may mask viable pathogens, supporting the adoption of culture-independent diagnostics such as polymerase chain reaction (PCR) and matrix-assisted laser desorption/ionization time-of-flight mass spectrometry (MALDI-TOF MS) for improved pathogen detection, particularly in patients with suspected prior antibiotic exposure. Furthermore, antimicrobial susceptibility testing algorithms must evolve to account for the presence of pre-existing ABS to avoid misinterpretation of resistance phenotypes.

Reconsideration of bacteriuria thresholds in the presence of pre-existing ABS

The conventional threshold for significant bacteriuria (≥10⁵ CFU/mL) may be inadequate in the presence of pre-existing ABS, as residual antibiotics can suppress bacterial growth and yield falsely low colony counts. In such cases, low-level ("insignificant") bacteriuria should not be dismissed without considering clinical history and symptoms. Diagnostic criteria must be re-evaluated to reflect these nuances, particularly in symptomatic patients with recent antibiotic exposure. Tailored interpretation, supported by clinical context and advanced diagnostics, is essential for accurate diagnosis and appropriate antimicrobial stewardship.

Limitations and future directions

The study’s reliance on culture-based methods for pre-existing ABS detection may have underestimated antibiotic residue levels. More sensitive techniques like liquid chromatography-mass spectrometry (LC-MS) could improve detection fidelity. Additionally, molecular characterization of resistance genes was not performed, limiting mechanistic insights into resistance patterns. As a single-center study, generalizability is limited, and recall bias regarding prior antibiotic use is a potential confounder. Nevertheless, the data provide critical insight into the interplay between pre-existing ABS, diagnostic outcomes, and AMR in CA-UTIs.

## Conclusions

This study highlights that pre-existing ABS in urine significantly affects culture-based diagnosis of CA-UTIs, often leading to false-negative results and altered pathogen profiles. Such interference is largely attributed to prior antibiotic exposure, commonly through self-medication or empirical prescribing, which contributes to sub-therapeutic urinary antibiotic levels.

Given these findings, routine assessment of recent antimicrobial use and incorporation of antimicrobial stewardship (AMS) strategies are essential, particularly in high-risk groups such as diabetic patients. Diagnostic refinement may include lowering the colony-forming unit (CFU) threshold from ≥10⁵ to ≥10³ CFU/mL in symptomatic individuals with suspected ABS exposure. Integrating advanced diagnostics such as matrix-assisted laser desorption/ionization time-of-flight mass spectrometry (MALDI-TOF MS) and molecular assays can further mitigate the limitations of ABS interference. Future research should focus on standardizing ABS detection methods and understanding their role in antimicrobial resistance dynamics.
